# Identification of the Beagle 2 lander on Mars

**DOI:** 10.1098/rsos.170785

**Published:** 2017-10-11

**Authors:** J. C. Bridges, J. Clemmet, M. Croon, M. R. Sims, D. Pullan, J.-P. Muller, Y. Tao, S. Xiong, A. R. Putri, T. Parker, S. M. R. Turner, J. M. Pillinger

**Affiliations:** 1 Leicester Institute for Space and Earth Observation, Department of Physics and Astronomy, University of Leicester, Leicester LE1 7RH, UK; 2 Airbus, Gunnels Wood Road, Stevenage SG1 2AS, UK; 3 Citizen Scientist; 4 Mullard Space Science Laboratory, Department of Space and Climate Physics, University College London, Holmbury St Mary RH5 6NT, UK; 5 Jet Propulsion Laboratory, 4800 Oak Grove Drive, Pasadena, CA 91109, USA; 6 School of Physical Sciences, The Open University, Walton Hall, Milton Keynes MK7 6AA, UK

**Keywords:** Beagle 2, Mars, HiRISE

## Abstract

The 2003 Beagle 2 Mars lander has been identified in Isidis Planitia at 90.43° E, 11.53° N, close to the predicted target of 90.50° E, 11.53° N. Beagle 2 was an exobiology lander designed to look for isotopic and compositional signs of life on Mars, as part of the European Space Agency Mars Express (MEX) mission. The 2004 recalculation of the original landing ellipse from a 3-sigma major axis from 174 km to 57 km, and the acquisition of Mars Reconnaissance Orbiter High Resolution Imaging Science Experiment (HiRISE) imagery at 30 cm per pixel across the target region, led to the initial identification of the lander in 2014. Following this, more HiRISE images, giving a total of 15, including red and blue-green colours, were obtained over the area of interest and searched, which allowed sub-pixel imaging using super high-resolution techniques. The size (approx. 1.5 m), distinctive multilobed shape, high reflectivity relative to the local terrain, specular reflections, and location close to the centre of the planned landing ellipse led to the identification of the Beagle 2 lander. The shape of the imaged lander, although to some extent masked by the specular reflections in the various images, is consistent with deployment of the lander lid and then some or all solar panels. Failure to fully deploy the panels—which may have been caused by damage during landing—would have prohibited communication between the lander and MEX and commencement of science operations. This implies that the main part of the entry, descent and landing sequence, the ejection from MEX, atmospheric entry and parachute deployment, and landing worked as planned with perhaps only the final full panel deployment failing.

## 1. Introduction

### 1.1. The Beagle 2 mission

**Figure 1.**
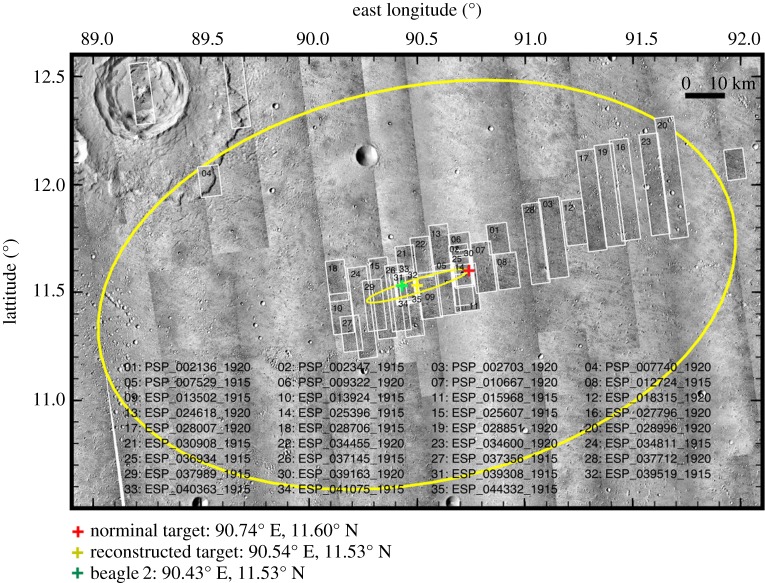
The Beagle 2 landing site. Image shows a Context camera on Mars Reconnaissance Orbiter (CTX) background with HiRISE thumbnails superimposed. The large ellipse is the 174 × 106 km ellipse at the time of launch in 2003. The smaller ellipse was recalculated 2004–2006 and is 57 × 7.6 km. The 35 HiRISE thumbnails around the final ellipse are numbered with their identifiers. Beagle 2 was identified at 90.43° E, 11.53° N, within about 20 km of the original, nominal target.

**Figure 2.**
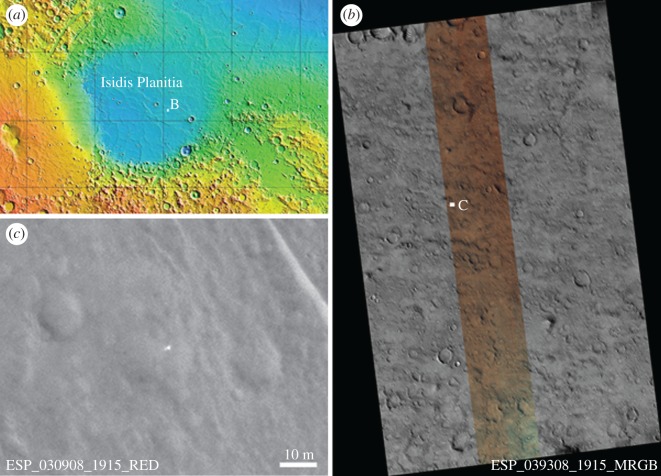
Location of the proposed Beagle 2 lander candidate. (*a*) MOLA map showing the Beagle 2 landing site and identified location (*b*) within Isidis Planitia, (*b*) HiRISE 0.25 m per pixel image ESP_039308_1915_MRGB showing colour coverage and lander position. This area was also used for follow-up searches for the lander after the initial identification (see §2.1.). (*c*) HiRISE image ESP_030908_1915_RED showing proposed lander as a bright object at the centre of view (Sun illumination is from the southwest).

Beagle 2 was the small (72.7 kg including entry and landing systems) UK-led probe which was part of European Space Agency's (ESA's) Mars Express (MEX) mission launched on 2 June 2003. Beagle 2 was named after *H.M.S. Beagle*, the ship Charles Darwin sailed on between 1831 and 1836, during which voyage he made many of the observations that led him to write *On the Origin of Species*. Beagle 2 was Europe's first Mars lander project [1]. The lander (approx. 33 kg) was designed to search for extinct life via isotopically fractionated organic material on and below (approx. 2 m) the surface using the gas analysis package (GAP) mass spectrometer and a stepped combustion-based system [2]. Sub-surface samples would have been obtained using the so-called ‘mole’, a hammering self-burying penetrometer-type mechanism [3]. It was also designed to study the inorganic chemistry and mineralogy of the landing site with its other instruments [2] and take multispectral imagery with PanCam [4]. GAP was also designed to search for methane within the atmosphere as a potential biomarker and science objectives included attempting *in situ* K-Ar age dating of samples [5]. The original, planned landing site in Isidis Planitia at 11.60° areocentric north latitude, 90.74° eastward longitude and –3367 m MOLA elevation (figures 1 and 2) was described in [6]. The 3-sigma landing ellipse size at the time of launch was 174 × 106 km with a 74.9° azimuth. This ellipse had an area of 14 500 km^2^ posing a considerable challenge to any attempt to locate the lander and associated hardware. The entry, descent and landing (EDL) design was based on concepts developed for the Huygens parachute landing on Titan and NASA's Pathfinder and Mars Exploration Rovers (MERs) [7,8], with adaptations and design changes as needed for Beagle 2 and MEX [9].

Beagle 2 was successfully ejected from MEX during its approach to Mars on 19 December 2003. All pre-ejection telemetry showed the probe to be in good health. Entry into the atmosphere was expected at 02.51.22 on 25 December 2003 with touchdown on the surface 8 min later with first data transmission from the surface via NASA's Mars Odyssey spacecraft expected at 05.25 [10]. No communication with Beagle 2 was possible until successful deployment of all four solar panels (§1.2) and after four months of attempts at communication (24 in total) via both Mars Odyssey and MEX, the lander, unable to communicate with Earth, was assumed to be lost. No communication was possible during EDL as there was no Mars spacecraft in the correct position to receive any signal owing to MER entry operations and MEX orbital insertion. Hence, no such communication system was built for Beagle 2. The Lovell Radio Telescope, at Jodrell Bank, UK, which acted as a ‘direct to Earth’ listening station was also not visible during EDL although attempts were made to detect Beagle 2 surface transmissions on 25, 26 and 27 December 2003 using the telescope. The last of these attempts were made in conjunction with the Stanford radio telescope. The reasons for the loss have remained unclear. An ESA enquiry in 2004 [11] detailed a number of potential causes for the loss including failure of airbags, potential tangling with parachute during airbag bounces. The project also held its own internal enquiry and catalogued a number of potential causes for the loss and produced a lessons-learnt document [12]. However, owing to lack of information it was not possible to eliminate any of the many potential causes.

In 2004, the landing ellipse size was recalculated—using Monte Carlo simulations and more accurate atmospheric entry coordinates [13,14] derived from MEX data—with a semi-major axis of 28.5 km and a semi-minor axis of 3.8 km, and this made identifying Beagle 2 on the Isidis ground surface more feasible with High Resolution Imaging Science Experiment (HiRISE) (figures 1 and 2). Isidis is a generally flat plain with moderate topography and relatively few slopes greater than a few degrees [6]. The positive surface features, e.g. figure 2, are surface expressions of relict craters, possible tuff cones and wrinkle ridges. The partial infilling of some larger kilometre-size craters was interpreted as a result of an Amazonian sedimentary influx across the basin's underlying Hesperian volcanic basement [6]. The generally subdued topography means that identifying the Beagle 2 lander (example of the HiRISE images used in figure 2) was feasible. HiRISE is a 25 cm per pixel resolution camera with 400–1000 nm red : blue-green colour capability onboard the Mars Reconnaissance Orbiter, which has a 255 × 300 km orbit [15]. It has been in operation since 2006 and using this high resolution and colour, previous landers have been located on the surface: Viking 1 and 2 (1976), Pathfinder (1996), MER Spirit and Opportunity (2004), Phoenix (2008), and Mars Science Laboratory (2012).

In this paper, we describe the Beagle 2 lander deployment and report the results of a search for it and its parachute, front heat shield and rear cover, in HiRISE images and high resolution image products derived from them. Based on the results, we outline an explanation of what occurred during landing in December 2003.

### 1.2. Beagle 2 entry descent and landing

**Figure 3.**
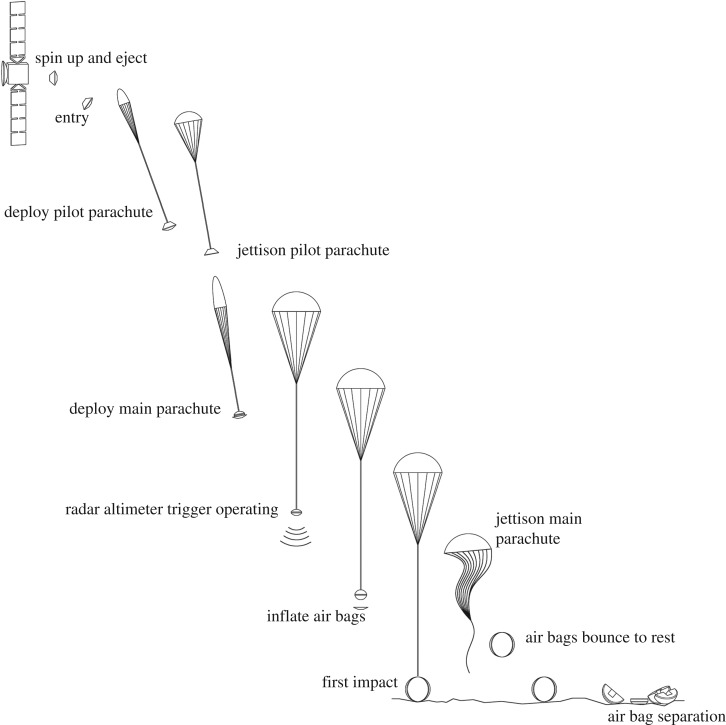
Beagle 2 entry, descent and landing sequence. Spin and up and eject (from Mars Express) to entry took 6 days. The pilot parachute pulling away the back cover and releasing the main parachute occurred at 2.6 km. Air bags were inflated at an altitude of 280 m. From atmospheric entry, at a closing velocity of 6 km^−1^, to impact and airbag separation took 8 min. Local time at landing was approximately midday.

The initial phase of the descent trajectory through the Mars atmosphere was planned based on aerodynamic deceleration of the probe from 5.6 km s^−1^ (figure 3). Following pilot parachute deployment, the predicted speed decreased from supersonic to subsonic values. The front heat shield and back cover were then released, with the pilot parachute pulling away the back cover, releasing the main parachute at 2.6 km altitude, 94 m s^−1^. The planned landing sequence then commenced with radar altimetry triggering inflation of the airbags at approximately 280 m above the ground. The airbags were made of three layers of polyester woven fabric and inflated with ammonia to a pressure of 100 mbar compared to the Martian atmosphere pressure of approximately 7 mbar. Ammonia was chosen for its liquefied storage performance and to avoid contaminating the surface with organic compounds following the expected leakage of the gas during and after landing. The outer two layers were designed to act as abrasion layers. First impact on the ground occurred up to 7 min after atmospheric entry. Once stationary, the airbags should have been released with the lander falling approximately 1 m to the Martian surface. The on-surface deployment sequence then started with the opening of the lander followed by solar panel deployment.

### 1.3. Beagle 2 hardware and solar panel deployment sequence

**Figure 4.**
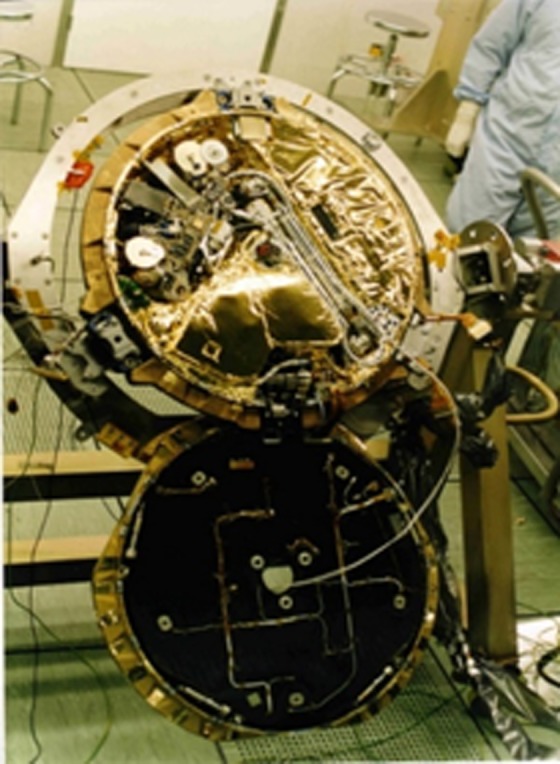
Beagle 2 lander in the Airbus, Stevenage, UK construction facility prior to integration with Mars Express. The instruments within the base are visible; the solar panels are stowed in the lid. The base is approximately 0.7 m in diameter.

**Figure 5.**
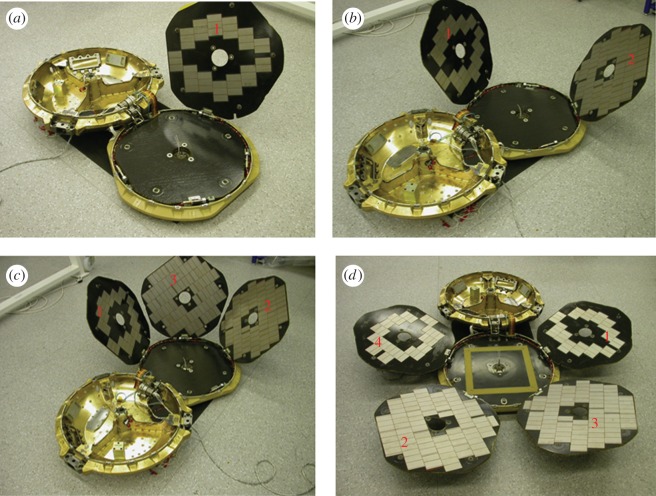
Beagle 2 model showing solar panels 1–4, then the base deployment. Each panel is 0.57 m in diameter, and the fully deployed lander diameter (*d*) is 1.7 m across. The fully deployed panels—as they were planned for Martian deployment—are at an angle of 20° to the horizontal. See text for more details on panel deployment. Other features visible in (*d*) include the UHF antenna which is only exposed upon deployment of all the panels.

To aid interpretation of the HiRISE images, it is important to first understand how the hardware may appear on the Martian surface, the physical sizes, and particularly surface reflectivity and colour. With a successful landing and deployment of Beagle 2 on the surface of Mars, examination of orbiter images of the landing ellipse would have been focused on looking for four main objects: the lander itself, the main parachute, the rear cover with the pilot chute attached and the front heat shield. Each of those is sufficiently distinguishable from the natural terrain to make their discovery possible. The airbag segments are likely to present a greater challenge because of their tan colour. In the context of searching for the elements of Beagle 2 on the surface of Mars, it should be noted that, with the exception of the main parachute (10 m canopy), the largest dimension—the front heat shield diameter—is 0.93 m, and the maximum possible dimension of the lander with all solar panels deployed is 1.7 m (figures 4 and 5). This is to be compared with a HiRISE pixel size of 0.3 m.

In more detail, the lander is comprised a shallow bowl 0.66 m in diameter and 0.08 m, a lid of the same diameter but only 0.03 m deep, and four pentagon-shaped solar panels nominally 0.57 m across. The panels were stowed in a stack in the lid with their rear surface uppermost. This is shown in figure 5. All structural elements were constructed from carbon fibre reinforced plastic composite, but the reasonably reflective resin-rich surface was not always exposed. The front surface of each solar panel has 85% solar cell coverage providing a highly reflective surface when deployed. Solar cells are designed to absorb energy from the red part of the spectrum, and thus a low red : blue-green spectral ratio signature is expected, and is one of the key criteria used in identifying the lander on the Martian surface. With all panels deployed, the UHF antenna embedded into the lid would be exposed (figure 5*d*). The surface of the lid is a carbon fibre composite with a reflective resin-rich surface.

The gold-coated Kapton thermal blankets of the base provide a highly reflective specular finish and account for nearly two-thirds of the exposed base following lid deployment. The remaining one-third is accounted for by the robotic arm wrapped in aluminized tape and the instruments' predominantly aluminium in composition. The back surface of each of the solar panels is carbon fibre composite but has the texture of the bleed cloth imprinted into it from the manufacturing process. Therefore, although resin-rich, these surfaces provide a more diffuse reflectivity. This would have been the surface presented by the lid if not all panels were deployed, with the reflectivity dominated by the gold-coated blanket and the number of deployed solar panels themselves.

After operating the hold-down devices to release the solar panel stack, the lander software would have autonomously commanded the deployment of each of the four solar panels in turn through a series of angle thresholds. Obstruction monitoring would have been initiated; an illumination check is performed at 130°. If this proved negative, deployment would have continued through to a maximum initial angle of 160°. If a panel failed to deploy to at least 130°, the deployment would have timed out with the deployment of the next panel commanded, and so on to full deployment of the four panels.

**Figure 6.**
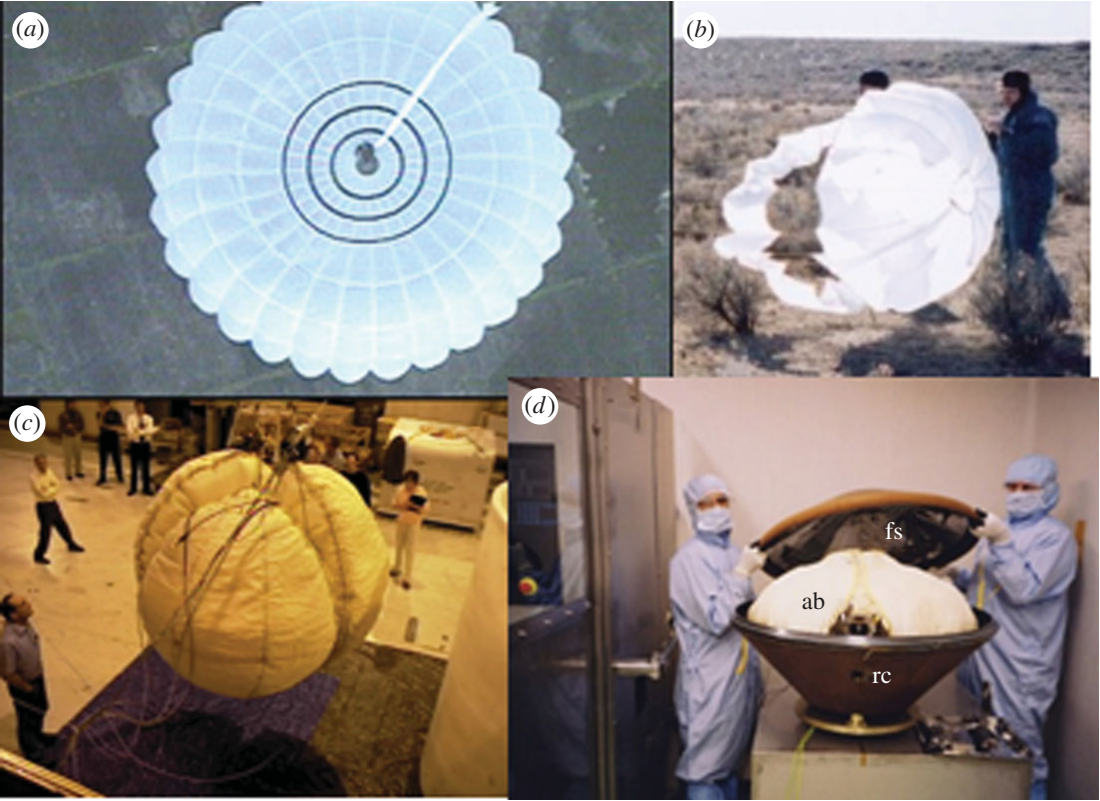
(*a*) Main parachute (10 m diameter) imaged during a parachute test (see [9] for details). (*b*) Pilot parachute testing in New Mexico, 2002. (*c*) Airbag composed of three segments–during drop testing at Johnson Space Center, 2002. (*d*) Probe aeroshell during vibration testing at Atomic Weapons Research Establishment, UK 2003. In addition to the lander itself, the front shield (fs) and rear cover (rc) may have been identified in HiRISE images; note the fs is fitted with an inner highly reflective aluminized Kapton multilayer insulation; ab, airbags.

The main parachute (figure 6*a*) would have been released from the lander at the point of first impact and would remain close to this impact zone. The parachute was fabricated [9] from a white translucent nylon fabric with a 10 m diameter when fully inflated. However, the parachute was expected to collapse on the surface in a more compact shape of the order of 3–4 m.

At the initial contact with the ground surface, following a first bounce predicted to be less than 100 m, the airbags would have continued to bounce along the descent trajectory to a total distance of up to 500 m depending on descent rate, wind, terrain and airbag pressure [9]. Conditions at the time of landing would have resulted in significantly lower values. Once at rest and after the airbag lacing was cut and the gas (ammonia) generator used for airbag inflation was released, the strain energy in the three airbag segments (figure 6*c*) would have caused each to be ejected away from the lander as it fell to the surface. The distance travelled by each segment depended on their residual gas pressure and terrain topography and is predicted to be in the range 5–10 m from the lander, but with much uncertainty. In addition, the gas generator was tethered to one segment to enable it to be dragged clear of the lander. Each segment had a length of 1930 mm and a width of 965 mm when inflated (figure 6*c*). Airbag leakage would have resulted in the collapse of each segment. The outer layers of the airbag are constructed from a yellow/tan fabric with poor reflectivity characteristics.

The probe aeroshell (figure 6*d*) was composed of the front heat shield and the back cover. These share a carbon fibre composite construction with ablative tiles bonded to their exterior surfaces. During the early phase of atmospheric entry, these tiles were expected to see very high temperatures resulting in a burnt, black carbon surface. The back cover is a truncated cone with a maximum diameter of 0.93 m and a length of 0.31 m. The interior has a resin-rich surface. The front heat shield is a shallow cone 0.93 m in diameter with a depth of 0.225 m and is fitted with an inner highly reflective aluminized Kapton multilayer insulation blanket. Both were fitted with external black Kapton multilayer insulation for thermal control during the cruise and coast phases of the mission. These outer blankets were not expected to survive aero entry and either detached or burnt up. The pilot parachute was designed to remain attached to the back cover by a strop and rigging giving a maximum tether length of 8.5 m. The pilot parachute itself had an inflated diameter of 2 m and was manufactured from a white nylon fabric, but less translucent than the main chute (figure 6*b*).

## 2. The search for Beagle 2 hardware

**Figure 7.**
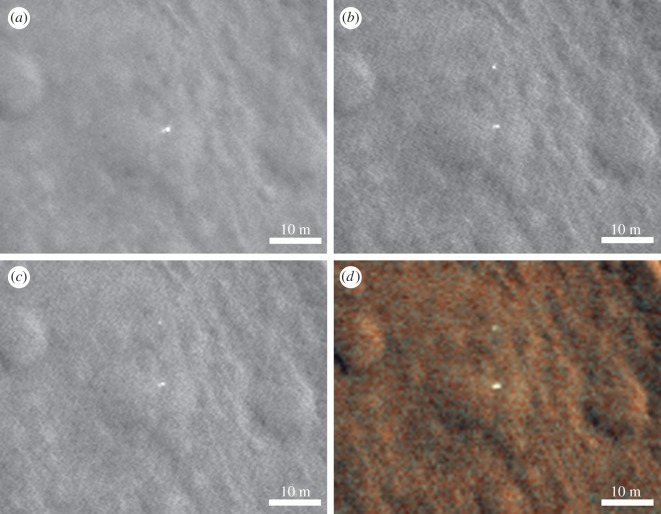
HiRISE images centred on the proposed lander and associated front shield, 12 m above. The illumination conditions are summarized in the electronic supplementary material. Sun illumination is from the southwest in each case. Pixel size is 0.25 m (*a,b,c*) and 0.5 m (*d*). For regional context see figure 2. (*a*) HiRISE ESP_030908_1915_RED; (*b*) HiRISE ESP_039519_1915_RED; (*c*) HiRISE ESP_039308_1915_RED; (*d*) HiRISE colour ESP_039308_1915_MRGB. The variation in appearance of the lander between different images in this figure is consistent with specular reflections.

A key stage in the search for Beagle 2 was the recalculation of the landing ellipse in 2004 based on the known MEX trajectory [13,14]. This produced a 3-sigma semi-major axis of 28.5 km and a semi-minor axis of 3.8 km, with an azimuth of 16.81° from east, for a nominal landing point of 90.50° E, 11.53° N [13], (figures 1, 2 and 7). This recalculation greatly reduced the surface area on Mars that needed to be imaged. The second key stage was the deployment of Mars Reconnaissance Orbiter in 2006 and its HiRISE camera with 0.30 m per pixel with 6 km swath at a 300 km altitude, a 0.25 m pixel size and 5 km swath at a 250 km altitude when all 10 red charge-coupled devices (CCDs) were in operation (there have been nine since 2011). Importantly for our study, HiRISE also has red : blue-green colour capability [15]. The search was performed on greyscale, non-map projected HiRISE products at their native resolution, using HiView as the viewing application. Between 2006 and June 2014, a total of 26 HiRISE images were examined for signs of Beagle 2 (figures 1 and 7). A third key stage was provided by one of the co-authors (M.C.) who identified a gap in HiRISE coverage. In response to his HiWish request for more imagery, ESP_037145_1915 was acquired on 29 June 2014. In November 2014, he noted possible identification of the lander in this image and requested repeat imaging of it.

All but five images of the general landing area we report on here were taken at the highest possible resolution, giving a pixel size of better than 30 cm per pixel. Each image covers a surface area 5 km wide and 8–25 km long and was searched by visual inspection. With image sizes ranging from 18 000 to 20 000 pixels in width and 30 000 to 90 000 pixels in height, the visible area on a computer screen fits small fractions (a few tenths of 1%) of the entire image only. Therefore, the visible screen area was manually scrolled horizontally and vertically until all parts of a given image were visited. This way a careful inspection of the images was made. We have shown the detailed summary in Excel table format, in the electronic supplementary material.

The four main parts of the Beagle 2 lander and EDL hardware that we have searched for in HiRISE images are: the lander itself with its solar panels, the main parachute, the front shield and the back cover with the pilot parachute. These were all expected to be within close proximity to each other at the landing site. Expected separations were up to hundreds of metres for the main parachute, rear cover (and pilot chute) and the lander, up to 10 m for the lander and airbags with front shield within 100–200 m of the lander based on Monte Carlo modelling of the aerodynamics and landing [9].

The first HiRISE image covering the identified landing site of Beagle 2 ESP_030908_1915 was taken in February 2013 (figure 7). We searched this image in February 2014 but overlooked the suggested EDL hardware, probably because ESP_030908_1915 is subject to reduced contrast as it was taken around Mars' perihelion passage and dust was abundant in the atmosphere. The proposed Beagle 2 lander in ESP_030908_1915 does, however, show a very high relative brightness, possibly owing to specular reflection of the sunlight from the solar arrays or multi-layer insulation covered base. The high noise level in ESP_030908_1915 also means that the reflected light was scattered and may have registered on more CCD pixels than would correspond to the actual size of Beagle 2 (figure 7).

**Figure 8.**
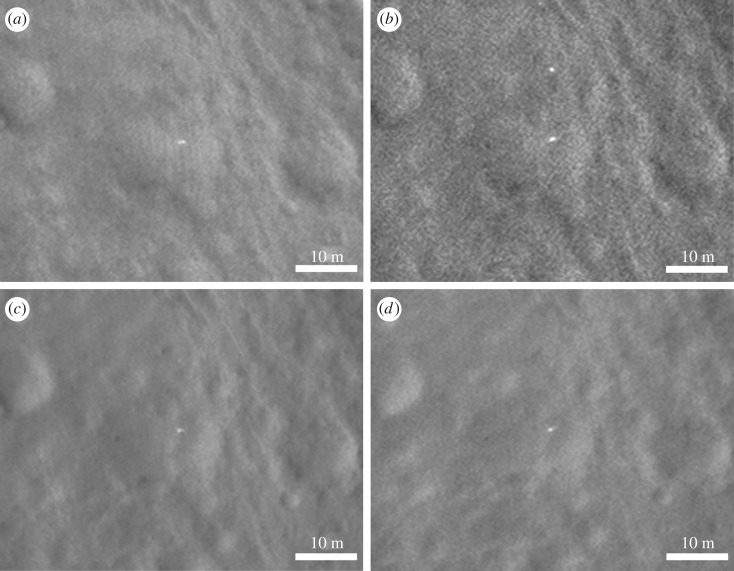
Further HiRISE images centred on the proposed lander and associated front shield 12 m above. Pixel size is 0.25 m in each case. Sun illumination is from the southwest (*a,b*) and from the northwest (*c,d*); see the electronic supplementary material for more details. For regional context see figure 2. (*a*) ESP_040363_1915_RED; (*b*) ESP_041075_1915_RED; (*c*) ESP_037145_1915_RED; (*d*) ESP_044332_1915_RED.

The second HiRISE image of the landing site is ESP_037145_1915, and was taken in June 2014 at the request of M.C. (figure 8). By the time we searched it in August 2014, we had thoroughly examined a total of 14 images. The noise level of ESP_037145_1915 is low (it was taken before the onset of the dust storm season). The suggested lander is much less striking in this image than in ESP_030908_1915 (figure 7), but still notable. Following this, we requested further HiRISE images over the objects of interest, giving colour and super-resolution and sub-pixel sampling capabilities.

**Figure 9.**
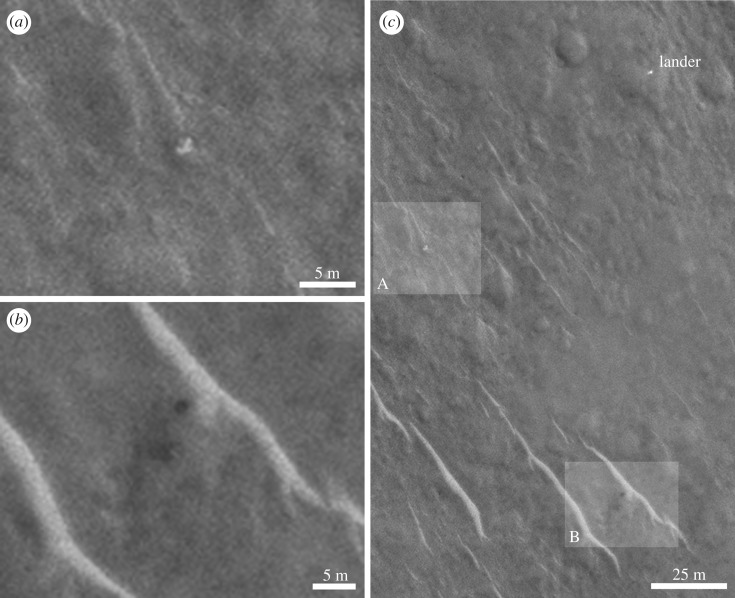
HiRISE images showing the proposed lander and nearby objects of interest studied in the search for parachutes and back cover. Source image is ESP_030908_1915_RED. Pixel size is 0.25 m and Sun illumination is from the southwest. (*a*) Light-toned, irregular, heterogeneous object in the centre of the field of view investigated as a possible parachute. (*b*) Dark-toned, geometric, homogeneous object investigated as a possible lander rear cover (figure 10 for more images of this object). (*c*) Proposed lander.

**Figure 10.**
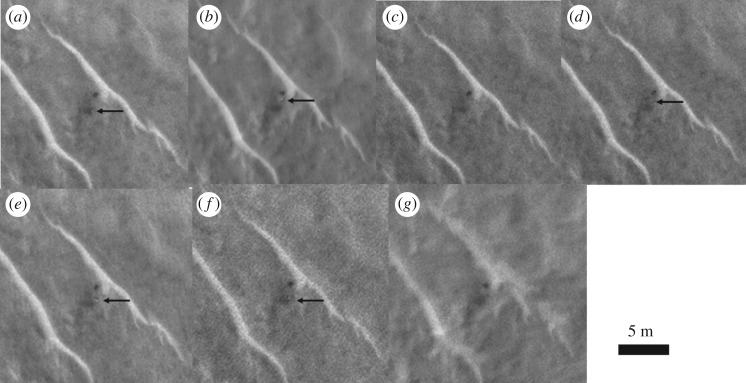
Beagle 2 rear cover candidate. The possible movement of the object, e.g. between (*a*) and (*b*), is consistent with a rear cover and parachute (figure 6). Rear cover candidate arrowed where visible. HiRISE images: (*a*) ESP_030908_1915_RED; (*b*) ESP_037145_1915_RED; (*c*) ESP_039308_1915_RED; (*d*) ESP_039519_1915_RED; (*e*) ESP_040363_1915_RED; (*f*) ESP_041075_1915_RED; (*g*) ESP_044332_1915_RED. See animated gif file in the electronic supplementary material.

In addition to searching for the lander itself, we searched for the other EDL components—notably the rear cover and its parachute and the main parachute. We studied candidate objects based on size and shape within a few hundred metres of the lander (figure 9). Examination in multiple and colour images led to all of these being without conclusive evidence. However, we suggest that the rear cover may also have been identified in some of the HiRISE images. This shows possible movement of an object between the different HiRISE images (see arrows in figure 10), though there is no red : blue-green data or morphology to give conclusive evidence.

### 2.1. Subsequent searches

Following the preliminary identification of the lander, two systematic searches for similar signatures within an 11 × 5 km region of the proposed lander, one based on greyscale value and one based on colour, were performed (see the electronic supplementary material). Scrutiny of each close-up portion of the search area ensured efficient interpretation and rejection of obvious natural features and known image artefacts. In addition to looking for lander-like objects, each search was also tuned to detect anomalous objects of similar scale based on tonal value (dark or bright), regularity of shape, homogeneity and context.

The first search was based on greyscale value and was conducted using the most suitable high-resolution red channel HiRISE image available (ESP_030908_1915_RED, 0.25 m per pixel). Figure 2*b* shows the search area and examples of the signatures used. The total area searched was approximately 55 km^2^ which equates to about 1 billion pixels. Other HiRISE images overlap the same area but were acquired at different times and under different illumination conditions, and these were used to verify whether potential matches were real or not (see the electronic supplementary material). Another search was conducted using the 0.5 m per pixel colour HiRISE RDR product ESP_039308_1915_MRGB (figure 2*b*) and the corresponding infrared, red and blue-green channels from the HiRISE EDR images.

It is clear that what we are suggesting is that the lander is not a detector artefact, e.g. noise or cosmic ray effects on the HiRISE CCD. The same object has been identified in multiple images under different illumination conditions. Furthermore, our searches did not reveal any similar objects.

### 2.2. Super high-resolution techniques for HiRISE imagery

**Figure 11.**
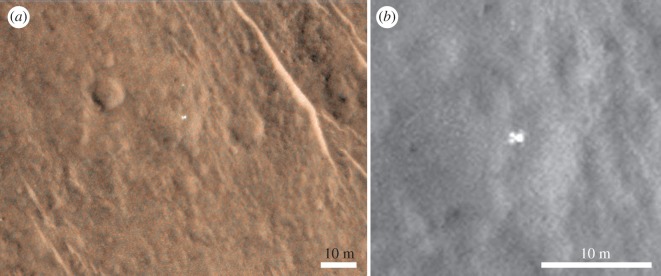
Super-resolution technique of lander, using images listed in table 1. This technique used ArcMap to georeference the images one to the other (see text for more details). (*a*) HiRISE MRGB ESP_039308_1915_MRGB showing lander and potential front shield 12 m above it. (*b*) Red channel ESP_039308_1915_MRGB with expanded view across the lander.

Following the identification of potential lander elements in the HiRISE images, we used two further techniques based on HiRISE imagery to characterize the proposed lander elements at sub-pixel resolution. The first SuperRes technique we employed (figure 11) made use of ArcMap to georeference the images (table 1), one to the other, in a manner similar to the base maps for MER Opportunity and Curiosity. We started with four tie points at the corners of the area of overlap between the two HiRISE images, and kept bisecting the area until mismatches were less than a couple of metres. Finally, we went to the area around the objects of interest and added an additional 110 tie points at rocks and other sharp brightness boundaries in the scene, but avoiding the hardware features so as not to pick a pixel that is bright in one image and not in the other, and tie it to the wrong reflector in the other image. A spline fit was used to help minimize residual distortions owing to the approximately 4° different emission angles (table 1) between the images causing small differences in the locations of rocks owing to topography. The result was an image pair that was accurately tied at sub-pixel scales over the hardware objects between tie points. Once satisfied with the registration, a cropped area around the objects was exported at 25 cm per pixel. With a number of images over the site, this process can enable a ‘manual super-resolution’ output. However, our experience shows that the improvement in resolution reaches a maximum of order 2 times, regardless of the number of images used.

**Table 1.** HiRISE images used in super-resolution georeference technique.

HiRISE images	emission angle	date captured
ESP_030908_1915	2.5°	28 February 2013
ESP_037145_1915	6.8°	29 June 2014
ESP_039308_1915	11.7°	15 December 2014
ESP_040363_1915	17.3°	7 March 2015
ESP_044332_1915	11.5°	10 January 2016

A second technique that we employed is a novel super-resolution restoration (SRR) technique, called Gotcha partial differential equation (PDE)-based total variation (TV) super-resolution restoration (GPT-SRR) [16], to be able to restore 5–12.5 cm pixel images from a stack of 25 cm pixel size HiRISE images. This technique employs the 5th generation of an adaptive least-squares correlation and region growing (Gotcha) image matcher [17] to derive extremely accurate sub-pixel ‘motion vectors’ (down to 0.01 pixels) and thus relate the information revealed from different orbiting view angles for the same feature/area to a higher-resolution grid. After this initial matching stage, a fourth-order PDE-based TV approach is used for stochastic refinement, subject to minimizing the modelled similarity and regularization cost. In addition, the restoration process applies a segmentation-based approach to keep the flat areas (with little multi-angle information available) smooth and feature-rich areas sharp (with multi-angle information available).

**Figure 12.**
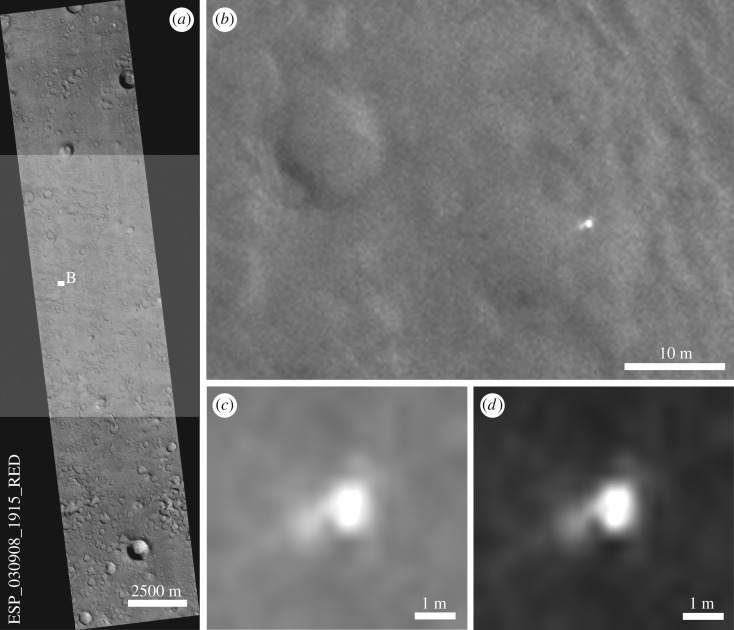
Subsequent search for lander-like signatures and super-high resolution. (*a*) Shaded portion shows area of image ESP_03908_1915_RED used for native resolution greyscale value search. (*b*) Close-up of lander object with 10 m crater for local value and morphological context (native resolution, default lander signature used for search). (*c*) Lander signature (contrast-stretched and simplified, un-normalized, used as the shape reference). (*d*) Lander signature (synthetic, normalized, used as the shape reference).

**Figure 13.**
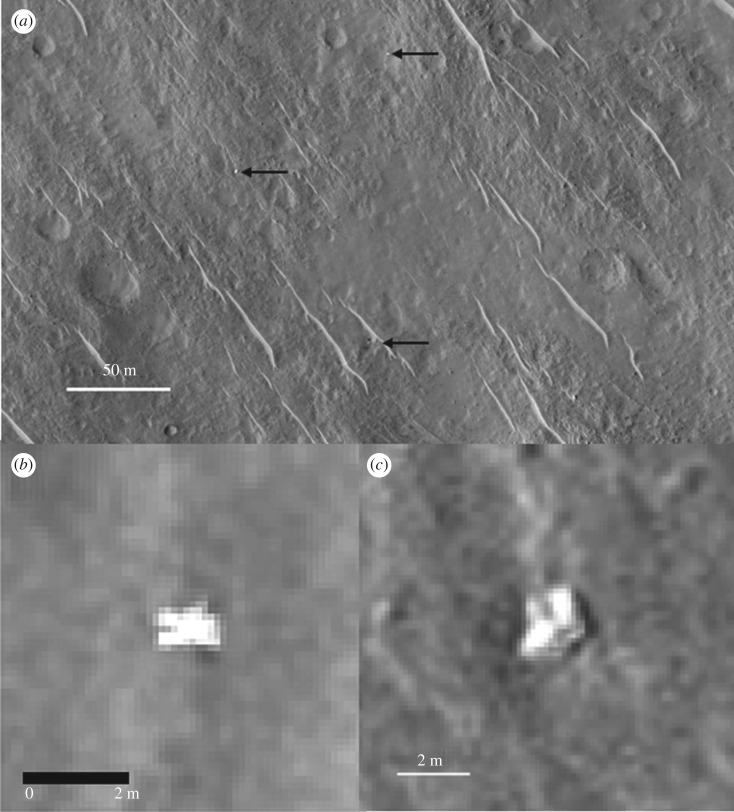
Super-resolution restoration technique in image (*a*) (lander, parachute candidate and rear cover candidate arrowed, figure 6) and zoomed-in view of the lander (*b*) and parachute target (*c*). See animated gif files in the electronic supplementary material.

From the SRR experiments performed to date over MER and Mars Science Laboratory rover traverses [16], multiple overlapping HiRISE input images (25 cm) are processed to generate up to a factor of 5 enhancement in resolution. Comparison of the original and SRR HiRISE images and rover Navcam orthorectified image mosaics [18] has demonstrated that these SRR results can reveal new information including the imaging of individual rocks (diameter ≥ 30 cm). Further SRR experiments were applied to the Beagle 2 lander elements, where GPT-SRR was applied to a stack of five HiRISE scenes captured to date since February 2013 covering the same area, enabling us to see more details of the potential lander (figures 12 and 13).

### 2.3. Brightness, red and blue-green values from colour HiRISE EDR and RDR images

As part of our characterisation of the lander and surrounding terrain we studied the relative brightness of the lander, and red and blue-green channels in a colour HiRISE EDR image (figure 7*d*). In addition to the uncalibrated EDR data we used an USGS ISIS procedure [19] to radiometrically calibrate the values (RDR in the electronic supplementary material). We found no conclusive differences in R : GB ratios for EDR or RDR products between the lander elements and local terrain. Partial dust cover and atmospheric dust are likely to mask colour variations [20]. However, the data do show large variations in overall brightness of the main lander element between images, and a high relative brightness compared to surrounding terrain (for example, figures 7,8,9,11). This is consistent with the surface reflectance, specular properties of the solar panel and other metallic lander surfaces, and the varying solar elevation/incidence angles of the HiRISE images. The surrounding natural terrain gives diffuse reflectance and, despite a systematic search, no objects of similarly high relative high brightness relative to the local terrain were found (electronic supplementary material).

### 2.4. Lander identified in the HiRISE images and landing scenario

The bright, 1.5 m diameter, multicomponent, object—identified as the lander—at 90.43° E, 11.53° N is apparently lying flat, as it does not cast a recognizable shadow in any of the images. The variation between images is consistent with specular reflection. However, imagery suggests that it is composed of at least four lobes (figures 12 and 13). This is consistent with partial or complete solar panel deployment. Within 12 m, to the north of the lander, the bright object that appears in the images, with red : blue-green ratio of 1.4 may be the front shield (figure 13*a*), which would be expected to be close to the lander. As shown in figure 10 and §2, and the electronic supplementary material animated GIFs, we also have a candidate object for the rear cover and its parachute.

**Figure 14.**
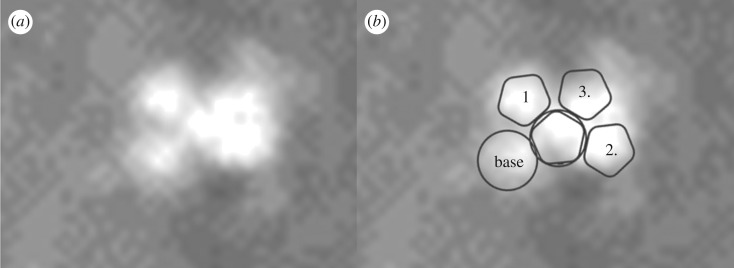
(*a*) Super-resolution sampling, close-up view of the lander based on HiRISE MRGB ESP_039308_1915_MRGB with super-resolution sampling (figure 13*b*)*.* (*b*) Beagle 2 potential deployment scenario with numbered solar panels 1–3, base and lid (figure 5 for context).

The identification of the intact lander suggests that following nominal ejection from MEX, atmospheric entry and descent occurred as planned. The lack of EDL telemetry data means that we cannot be certain that the nominal set of procedures as shown in figure 3 occurred as planned, but the intact nature of Beagle 2 suggests that it did until deployment of the solar panels. Possible failure to deploy all of the solar panels would have meant that the UHF transmitter's antenna (figure 5*d*) was obscured and thus communication between Beagle 2 and MEX was precluded. This in turn would have stopped any initial data being returned and operations commencing. In figure 14*b,* we show a potential solar panel deployment scenario consistent with the HiRISE and sub-pixel imagery, although we note that other possibilities are consistent with the images, e.g. partial if not complete deployment of the fourth panel. The variation in specular reflections between the different images makes an accurate deployment scenario problematic. However, at least the lid and solar panels 1–3 could have been deployed, and possibly all of the panels were deployed. Failure to complete panel deployment could have been caused by damage to the lander, e.g. wiring or mechanisms or software issues during landing. With all panels deployed, partially or otherwise, damage to the antenna system would also need to be included.

To precisely determine the exact coordinates of the lander, we have used a HiRISE-CTX-HRSC co-registration technique described in [21]. Using HiRISE ESP_039308_1915 with CTX D19_034455_1918_XN_11N269 W and with HRSCHh1193_0001, the exact co-ordinates are 90.43139° E, 11.52879° N.

## 3. Conclusion and implications

The location at 90.43° E, 11.53° N (more exactly 90.43139° E, 11.52879° N), 1.5 m size, multi-lobed shape, and high reflectivity relative to the surrounding natural terrain, with specular reflections, of an object identified in HiRISE imagery and sub-pixel-sampled imagery derived from them, suggest a high probability of having identified the Beagle 2 lander on the surface of Mars close to the planned target in Isidis Planitia. The success of the search benefited from three propitious circumstances. Firstly, the initial landing ellipse size was recalculated to a 3-sigma major axis length of 57 × 7.6 km from the at-launch values of 174 × 106 km. Secondly, the landing site was imaged at the full resolution of 30 cm per pixel by HiRISE from 2006 onwards. It would have been impossible to identify the Beagle 2 hardware at 60 cm per pixel (2 × 2 binning) or in Mars Orbiter camera images (1.5–2 m per pixel). Thirdly, the landing site happens to be in the overlap of two HiRISE images, which allowed us to cross-check the candidate against the other images. Systematic searches in HiRISE images in an 11 × 5 km region around the identified lander confirm the lander's unique identification compared to rocks and sand ripples.

It is expected that other parts of Beagle 2 hardware i.e. parachutes, front shield, airbag segments and rear cover would be present in the vicinity, and we have identified some candidates, including a front shield approximately 12 m from the lander and a rear cover within a few hundred metres of the lander. The most confident identification is that of the lander. A lack of spectral and shape or size criteria hinder fully confident identification of other Beagle 2 elements, though the apparent movement of an object between HiRISE images is consistent with a rear cover attached to its pilot parachute.

Beagle 2 was the UK's and ESA's first attempt at landing on Mars. The data analysed above show that the lander completed its descent and landing sequence, the most difficult and risky part, but was prevented from communicating with its planned orbiting relay spacecraft (NASA Odyssey and ESA's Mars Express) probably owing to an incomplete deployment, though that is yet to be fully proved. This shows that the engineering of the spacecraft was basically sound. Further analysis of the data and additional images of the landing site may elucidate the cause of the failure to communicate.

## Supplementary Material

Beagle 2 Appendix Search Study Characteristics

## Data accessibility

The data used in this paper are stored at data dryad: http://dx.doi.org/10.5061/dryad.707kc [22]. This is an Excel spreadsheet where the results of the search for Beagle 2, Mars coordinates, HiRISE imagery id, red : blue-green data, etc. are catalogued. The data also include the animated GIFs over the lander, rear cover and parachute candidates.
